# RETRACTION: The Impact of Glucocorticoids Therapy on Cutaneous Wounds in Kawasaki Disease: A Meta‐Analysis of Randomized Controlled Trials

**DOI:** 10.1111/iwj.70544

**Published:** 2025-04-18

## Abstract

**RETRACTION:** HuJ. , GaoL. , FuS. , WangW. , XieC. , ZhangY. , KeH. , and GongF. , “The Impact of Glucocorticoids Therapy on Cutaneous Wounds in Kawasaki Disease: A Meta‐Analysis of Randomized Controlled Trials,” International Wound Journal 21, no. 3 (2024): e14812, 10.1111/iwj.14812.38444059 PMC10915126

The above article, published online on 05 March 2024, in Wiley Online Library (http://onlinelibrary.wiley.com/), has been retracted by agreement between the journal Editor in Chief, Professor Keith Harding; and John Wiley & Sons Ltd. Following an investigation by the publisher, all parties have concluded that this article was accepted solely on the basis of a compromised peer review process. The editors have therefore decided to retract the article. The authors did not respond to our notice regarding the retraction.

**RETRACTION:** J. Hu , L. Gao , S. Fu , W. Wang , C. Xie , Y. Zhang , H. Ke , and F. Gong , “The Impact of Glucocorticoids Therapy on Cutaneous Wounds in Kawasaki Disease: A Meta‐Analysis of Randomized Controlled Trials,” International Wound Journal 21, no. 3 (2024): e14812, 10.1111/iwj.14812.38444059 PMC10915126

The above article, published online on 05 March 2024, in Wiley Online Library (http://onlinelibrary.wiley.com/), has been retracted by agreement between the journal Editor in Chief, Professor Keith Harding; and John Wiley & Sons Ltd. Following an investigation by the publisher, all parties have concluded that this article was accepted solely on the basis of a compromised peer review process. The editors have therefore decided to retract the article. The authors did not respond to our notice regarding the retraction.

